# Still No Free Lunches: The Price to Pay for Tighter PAC-Bayes Bounds

**DOI:** 10.3390/e23111529

**Published:** 2021-11-18

**Authors:** Benjamin Guedj, Louis Pujol

**Affiliations:** 1 Centre for Artificial Intelligence, Department of Computer Science, University College London, London WC1V 6LJ, UK; 2 Inria Lille—Nord Europe Research Centre and Inria London, 59800 Lille, France; 3 Laboratoire de Mathématiques d’Orsay, Université Paris-Saclay, CNRS, 91405 Orsay, France

**Keywords:** statistical learning theory, PAC-Bayes theory, no free lunch theorems

## Abstract

“No free lunch” results state the impossibility of obtaining meaningful bounds on the error of a learning algorithm without prior assumptions and modelling, which is more or less realistic for a given problem. Some models are “expensive” (strong assumptions, such as sub-Gaussian tails), others are “cheap” (simply finite variance). As it is well known, the more you pay, the more you get: in other words, the most expensive models yield the more interesting bounds. Recent advances in robust statistics have investigated procedures to obtain tight bounds while keeping the cost of assumptions minimal. The present paper explores and exhibits what the limits are for obtaining tight probably approximately correct (PAC)-Bayes bounds in a robust setting for cheap models.

## 1. Introduction

For the sake of clarity, we focus on the supervised learning problem. We collect a sequence of input–output pairs ${\left(X_{i},Y_{i}\right)}_{i=1}^{N}\in {\left(X\times Y\right)}^{N}$, which we assume to be *N* independent realisations of a random variable drawn from a distribution P on X×Y. The overarching goal in statistics and machine learning is to select a hypothesis *f* over a space F which, given a new input *x* in X, delivers an output f(x) in Y, hopefully close (in a certain sense) to the unknown true output *y*. The quality of *f* is assessed through a loss function *ℓ* which characterises the discrepancy between the true output *y* and its prediction f(x), and we define a global notion of risk as

$$R\left(f\right)=E_{(X,Y)∼P}\left[\ell \left(f(X),Y\right)\right].$$

The aim of machine learning is to find a good (in the sense of a low risk) hypothesis f∈F. In the generalised Bayes setting, the learning algorithm does not output a single hypothesis but rather a *distribution* ρ over the hypotheses space F and the associated bounds are called PAC-Bayesian bounds (see [1] for a survey of the topic).

As many probabilistic bounds stated in the statistics and machine learning literature, PAC-Bayesian bounds (where PAC stands for probably approximately correct—see [2]) commonly requires strong assumptions to hold, such as sub-Gaussian behaviour of some random variables. These assumptions can be misleading when dealing with true data as they do not take into account some practical situations, such as outlier contamination. Many efforts have been made recently to keep tight generalisation bounds valid with a few set of assumptions about the underlying distribution: this is known as robust learning [see [3] for a survey of the topic].

In this work we explore the possibility to establish a connection between recent techniques introduced by robust machine learning and PAC-Bayesian generalisation bounds. The result of our work is negative as we were not able to prove a PAC-Bayes bound in a robust statistics setting. However, we found it useful to write down our findings in order to give the interested reader a review of material involved in both robust statistics and PAC-Bayes theory and present the fundamental issues we faced as we believe it to be useful to the community.

**Organisation of the paper.** We introduce an elementary example and set a basic notation to illustrate the problem of robustness in Section 2, before providing an overview of recent advances in robust statistics in Section 3, and briefly introduce the field of PAC-Bayes learning in Section 4. We then propose in Section 5 a detailed study of the structural limits which do not allow for PAC-Bayes bounds which are simultaneously tight without requiring strong assumptions. The paper closes with a discussion in Section 6.

## 2. About the “No Free Lunch” Results

A class of results in statistics is known as “no free lunch” statements [see [4], Chapter 7]. The “no free lunch” results typically state that if one does not consider the restrictions on the modelling of the data-generating process, one cannot obtain meaningful deviation bounds in a non-asymptotic regime. The well-known trade-off is that the more restrictive the assumptions, the tighter the bounds. Let us illustrate this classical phenomenon by a simple example.

Assume that we have a dataset consisting in *N* real observations $x_{1},\cdots ,x_{N}\in R$ and consider they are independent, identically distributed (iid) realisations of a random variable *X*. Our goal is to estimate the mean of *X* and build a confidence interval for this estimate. As a start, let us focus on the empirical mean, denoted by $\bar{x}=\frac{1}{N}\sum_{i=1}^{N}x_{i}$. As “no free lunch” results state, we have to consider a class of distributions to which the data-generating distribution P belongs.

### 2.1. Expensive and Cheap Models

If there is always a price to pay in order to derive insightful result, there is a variety of degrees of restrictions. In the remainder of the paper, we will focus on two classical models corresponding to a different level of demand on the random variables.

A first type of restriction we can make is an “expensive modelling”. For σ>0, let $P_{\mathrm{expensive}}^{\sigma }$ be the set of all real-valued random variables *X* satisfying:

$$\log\left(E\left[\exp\left\{\lambda (X-E[X])\right\}\right]\right)\leq \frac{\lambda^{2}\sigma^{2}}{2}.$$

This $P_{\mathrm{expensive}}^{\sigma }$ is the class of sub-Gaussian random variables with variance factor $\sigma^{2}$ [see [5] for a complete coverage of the topic]. We call this model “expensive” as this restriction is often considered unrealistic for real-life datasets and is hard or impossible to check in practice.

An alternative type of restriction is a “cheap modelling”. For σ>0, let $P_{\mathrm{cheap}}^{\sigma }$ be the set of real-valued random variables with a finite variance, upper bounded by $\sigma^{2}$. We call this model “cheap” as this is considerably less restrictive than the expensive one and is much more likely to hold in practice.

### 2.2. Confidence Interval for the Empirical Mean

**Proposition** **1** (Confidence intervals)**.** *If we assume that $X\in P_{\mathrm{expensive}}^{\sigma }$, then for all δ∈(0,1/2), the following random interval is a confidence interval for the mean of X at level 1−δ:*

(1) $$\left[\;\;\bar{x}\pm \frac{\sigma }{\sqrt{N}}\sqrt{2}\times \sqrt{2\log\left(\frac{1}{\delta }\right)}\;\;\right].$$

*If we assume that $X\in P_{\mathrm{cheap}}^{\sigma }$, then for all δ∈(0,1), the following random interval is a confidence interval for the mean of X at level 1−δ:*

(2) $$\left[\;\;\bar{x}\pm \frac{\sigma }{\sqrt{N}}\sqrt{\frac{1}{\delta }}\;\;\right].$$

*In the case of a cheap model, there is no hope to obtain a significantly tighter confidence interval with respect to δ if one uses the empirical mean [as proved in [6], Proposition 6.2].*

**Proof.**  To establish the first confidence interval (1), we first remark that if $X\in P_{\mathrm{expensive}}^{\sigma }$, then $\bar{x}\in P_{\mathrm{expensive}}^{\sigma /\sqrt{N}}$ and $E\left[\bar{x}\right]=E\left[X\right]$. So, applying Theorem 2.1 of [5] to $\bar{x}-E\left[X\right]$ we obtain, for all a>0:

$$\begin{array}{cc} P\left(|\bar{x}-E\left[X\right]|>a\right) & =P\left(\bar{x}-E\left[X\right]>a\right)+P\left(\bar{x}-E\left[X\right]<-a\right)\\ \; & \leq 2\max\left(P\left(\bar{x}-E\left[X\right]>a\right),P\left(\bar{x}-E\left[X\right]<-a\right)\right)\\ \; & \leq 2\exp\left(-\frac{Na^{2}}{2\sigma^{2}}\right). \end{array}$$

Setting $\delta =\exp\left(-\frac{Na^{2}}{2\sigma^{2}}\right)$ leads to the expected result. The second confidence interval (2) is obtained through Chebychev’s inequality. $E\left[\bar{x}\right]=E\left[X\right]$ and as $X\in P_{\mathrm{cheap}}^{\sigma }$, $\mathrm{Var}\left(\bar{x}\right)=\frac{\mathrm{Var}(X)}{N}\leq \frac{\sigma^{2}}{N}$. So for all a>0

$$P\left(|\bar{x}-E\left[X\right]|>a\right)\leq \frac{\sigma^{2}}{Na^{2}}.$$

Now, setting $\delta =\frac{\sigma^{2}}{Na^{2}}$ we get

$$P\left(|\bar{x}-E\left[X\right]|>\frac{\sigma }{\sqrt{N}}\sqrt{\frac{1}{\delta }}\right)\leq \delta .$$

□

**Figure 1:**
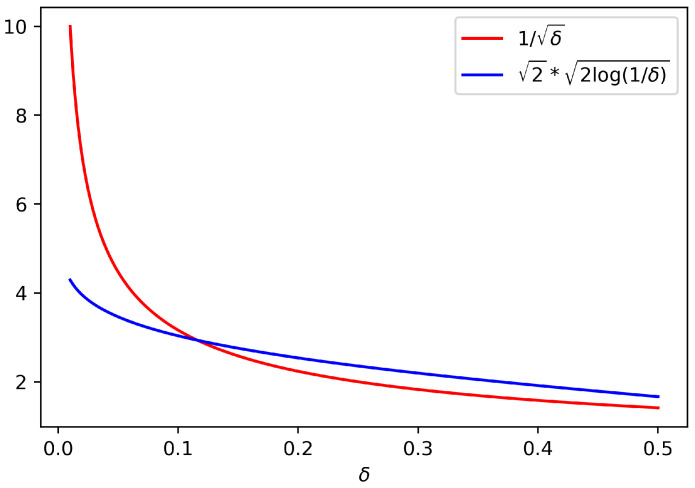
$\sqrt{2}\times \sqrt{2\log(1/\delta )}$ and $1/\sqrt{\delta }$ with respect to δ.

Note that the dependence in δ is fairly different in both confidence intervals defined in (1) and (2): for fixed $\sigma^{2}$ and *N*, the $\sqrt{2}\times \sqrt{2\log(1/\delta )}$ regime (following the lunch metaphor, the “good lunch”) is much more favourable than the $1/\sqrt{\delta }$ regime (the “bad lunch”). We illustrate this in Figure 1, where we plot $\sqrt{2}\times \sqrt{2\log(1/\delta )}$ and $1/\sqrt{\delta }$ as a function of δ∈(0,1/2). We remark that for small values of δ, corresponding to a higher confidence level, the interval (1) will be much tighter than (2).

So, while it is clear that the best confidence interval requires more stringent assumptions, there have been attempts at relaxing those assumptions—or in other words, keeping equally good lunches at a cheaper cost.

## 3. Robust Statistics

Robust statistics address the following question: can we obtain tight bounds with minimal assumptions—or in other words, can we get a good cheap lunch? In the mean estimation case hinted in Section 2, the question becomes the following: if $P\in P_{\mathrm{cheap}}^{\sigma }$, can we build a confidence interval at level 1−δ with a size proportional to $\frac{\sigma }{\sqrt{N}}\sqrt{2\log(1/\delta )}$?

As mentioned above, there is no hope to achieve this goal with the empirical mean. Different alternative estimators have thus been considered in robust statistics, such as M-estimators [6] or median-of-means (MoM) estimators [see [7] for a recent survey, and references therein].

The key idea of MoM estimators is to achieve a compromise between the unbiased but non-robust empirical mean and the biased but robust median. As before, let us consider a sample of *N* real numbers $x_{1},\cdots ,x_{N}$, assumed to be an iid sequence drawn from a distribution P. Let K≤N be a positive integer and assume for simplicity that *K* is a divisor of *N*. To compute the MoM estimator, the first step consists of dividing the sample $\left(x_{1},\cdots ,x_{N}\right)$ into *K* non-overlapping blocks $B_{1},\cdots ,B_{K}$, each of length N/K. For each block, we then compute the empirical mean

$${\bar{x}}_{B_{i}}=\frac{K}{N}\sum_{j\in B_{i}}x_{j}.$$

The MoM estimator is defined as the median of those means:

$${\mathrm{MoM}}_{K}\left(x_{1}\cdots ,x_{N}\right)=\mathrm{median}\left\{{\bar{x}}_{B_{1}},\cdots ,{\bar{x}}_{B_{K}}\right\}.$$

This estimator has the following nice property.

**Proposition** **2** ([7], Proposition 12)**.** *Assume $P\in P_{\mathrm{cheap}}^{\sigma }$, for $\delta =\exp\left(-\frac{K}{8}\right)$,*

(3) $$\left[{\mathrm{MOM}}_{K}\pm \frac{\sigma }{\sqrt{N}}\times 4\sqrt{2\log\left(\frac{1}{\delta }\right)}\right]$$

*is a confidence interval for the mean of X at the level 1−δ.*

This property is quite encouraging, as for a cheap model we obtain a confidence interval similar, up to a numerical constant, to the best one (1) in Section 2. However, we also spot here an important limitation. The confidence interval (3) for MoM is only valid for the particular error threshold $\delta =\exp\left(-K/8\right)$, which depends on the number of blocks *K* (a parameter for the estimator ${\mathrm{MoM}}_{K}$). The estimator must be changed each time we want to evaluate a different confidence level.

An ever more limiting feature is that the error threshold δ is constrained and cannot be set arbitrarily small, as in (1) or (2). Obviously, the number of blocks cannot exceed the sample size *N*, and the error threshold reaches its lowest tolerable value $\exp\left(-N/8\right)$. In other words, the interval defined in (3) can have confidence at most $1-\exp\left(-N/8\right)$.

Is this strong limitation specific to MoM estimators? No, say [8], [Theorem 3.2 and following remark]. This limitation is universal; over the class $P_{\mathrm{cheap}}^{\sigma }$, there is no estimator $\hat{x}$ of the mean such that there exists a constant L>1 such that

$$\left[\hat{x}\pm \frac{\sigma }{\sqrt{N}}\times L\sqrt{2\log\left(\frac{1}{\delta }\right)}\right]$$

is a confidence interval at level 1−δ for δ lower than $e^{-O(N)}$.

To sum up, a good and cheap lunch is possible, with the limitation that the bound is no longer valid for all confidence levels.

## 4. PAC-Bayes

We now briefly introduce the generalised Bayesian setting in machine learning, and the resulting generalisation bounds, the PAC-Bayesian bounds. PAC-Bayes is a sophisticated framework to derive new learning algorithms and obtain (often state-of-the-art) generalisation bounds, while maintaining probability distributions over hypotheses; as such, we are interested in studying how PAC-Bayes is compatible with good and cheap lunches. We refer the reader to [1,9] and the many references therein for recent surveys on PAC-Bayes including historical notes and main bounds. We focus on classical bounds from the PAC-Bayes literature, based on the empirical risk as a risk estimator—and we instantiate those bounds in two regimes matching the “expensive” and “cheap” models introduced in Section 2.

### 4.1. Notation

For any f∈F, we define the empirical risk $R_{N}\left(f\right)$ as:

$$R_{N}\left(f\right)=\frac{1}{N}\sum_{i=1}^{N}\ell \left(f\left(X_{i}\right),Y_{i}\right).$$

In the following, we consider integrals over the hypotheses space F. To keep the notation as compact as possible, we will write μ[g]=∫gdμ if μ is a measure over F and g∈F a μ-integrable function.

### 4.2. Generalised Bayes and PAC Bounds

The main advantage of PAC-Bayes over deterministic approaches which output single hypotheses (through optimisation of a particular criterion such as in model selection, etc.) is that the distributions allow us to capture uncertainty on hypotheses, and take into account correlations among possible hypotheses.

Denoting by ρ the posterior distribution, the quantity to control is:

$$\rho \left[R\right]=\int_{F}R\left(f\right)d\rho \left(f\right)$$

which is an aggregated risk over the class F and represents the expected risk if the predictor *f* is drawn from ρ for each new prediction. The distribution ρ is usually data-dependent and is referred to as a “posterior” distribution (by analogy with Bayesian statistics). We also fix a reference measure π over F, called the “prior” (for similar reasons). We refer to [1,10] for in-depth discussions on the choice of the prior: a recent streamline of work has further investigated the choice of data-dependent priors [11,12,13,14].

The generalisation bounds associated to this setting are known as “PAC-Bayesian” bounds, where PAC stands for probably approximately correct. One important feature of PAC-Bayes bounds is that they hold true for any prior π and posterior ρ. In practice, bounds are optimised with respect to ρ and possibly π. In the following, we focus on establishing bounds for any choice of π and ρ and do not mean to optimise.

### 4.3. Notion of Divergence

An important notion used in PAC-Bayesian theory is the divergence between two probability distributions [see [15], for example, for a survey on divergences]. Let E be a measurable space and μ and ν two probability distributions on E. Let *f* be a non-negative convex function defined on $R_{+}$ such that f(1)=0, we define the *f*-divergence between μ and ν by

$$D_{f}\left(\mu ,\nu \right)=\left\{\begin{array}{cc} \int f\left(\frac{d\mu }{d\nu }\right)d\nu & \mathrm{if}\;\mu \ll \nu ,\\ +\infty & \mathrm{otherwise}. \end{array}\right.$$

Note that we also use the notation *f* to denote hypotheses elsewhere in the paper, but we believe the context to always be clear enough to avoid ambiguity.

Applying Jensen inequality, we have that $D_{f}\left(\mu ,\nu \right)$ is always non-negative and equal to zero if and only if μ=ν. The class of *f*-divergences includes many celebrated divergences, such as the Kullback–Leibler (KL) divergence, the reversed KL, the Hellinger distance, the total variation distance, $\chi^{2}$-divergences, α-divergences, etc. Most PAC-Bayesian generalisation bounds involve the KL divergence.

A divergence can be thought of as a transport cost between two probability distributions. This interpretation will be useful for explaining PAC-Bayesian inequalities, where the divergence plays the role of a complexity term. In the following, we will just use two types of divergence. The first is the Kullback–Leibler divergence and corresponds to the choice f(x)=xlogx, which we denote it by

$$\mathrm{KL}\left(\mu ,\nu \right)=\left\{\begin{array}{cc} \int \log\left(\frac{d\mu }{d\nu }\right)d\mu & \mathrm{if}\;\mu \ll \nu ,\\ +\infty & \mathrm{otherwise}. \end{array}\right.$$

The second is linked to Pearson’s $\chi^{2}$-divergence and corresponds to the choice $f\left(x\right)=x^{2}-1$. It is referred to as $D_{2}$:

$$D_{2}\left(\mu ,\nu \right)=\left\{\begin{array}{cc} \int {\left(\frac{d\mu }{d\nu }\right)}^{2}d\nu \;-1 & \mathrm{if}\;\mu \ll \nu ,\\ +\infty & \mathrm{otherwise}. \end{array}\right.$$

To illustrate the behaviour of these two divergences, consider the case where μ and ν are normal distributions on $R^{d}$.

**Proposition** **3.** *If $E=R^{d}$, μ=N(a,I), and ν=N(0,I) (where I stands for the d×d identity matrix), we have*

$$\left\{\begin{array}{c} D_{2}\left(\mu ,\nu \right)=e^{{∥a∥}^{2}}-1,\\ \mathrm{KL}\left(\mu ,\nu \right)=\frac{1}{2}{∥a∥}^{2}. \end{array}\right.$$

**Proof.**  We have:

$$\left\{\begin{array}{c} d\mu \left(x\right)=\frac{1}{{\left(2\pi \right)}^{d/2}}\exp\left(-\frac{1}{2}{\left(x-a\right)}^{T}\left(x-a\right)\right)dx,\\ d\nu \left(x\right)=\frac{1}{{\left(2\pi \right)}^{d/2}}\exp\left(-\frac{1}{2}x^{T}x\right)dx,\\ \frac{d\mu }{d\nu }\left(x\right)=\exp\left(-\frac{1}{2}\left[-2x^{T}a+a^{T}a\right]\right)=\exp\left(-{∥a∥}^{2}/2\right)\exp\left(x^{T}a\right). \end{array}\right.$$

Then:

$$\begin{array}{cc} D_{2}\left(\mu ,\nu \right) & =\exp\left(-{∥a∥}^{2}\right)\int \exp\left(2x^{T}a\right)\frac{1}{{\left(2\pi \right)}^{d/2}}\exp\left(-\frac{1}{2}x^{T}x\right)dx-1\\ \; & =\exp\left(-{∥a∥}^{2}\right)\int \frac{1}{{\left(2\pi \right)}^{d/2}}\exp\left(-\frac{1}{2}x^{T}x+2x^{T}a\right)dx-1\\ \; & =\exp\left(-{∥a∥}^{2}\right)\exp\left({2∥a∥}^{2}\right)\int \frac{1}{{\left(2\pi \right)}^{d/2}}\exp\left(-\frac{1}{2}{\left(x-2a\right)}^{T}\left(x-2a\right)\right)dx-1\\ \; & =e^{{∥a∥}^{2}}-1. \end{array}$$

And finally:

$$\begin{array}{cc} \mathrm{KL}(\mu ,\nu ) & =\int \left(-\frac{{∥a∥}^{2}}{2}+x^{T}a\right)\frac{1}{{\left(2\pi \right)}^{d/2}}\exp\left(-\frac{1}{2}{\left(x-a\right)}^{T}\left(x-a\right)\right)dx\\ \; & =-\frac{{∥a∥}^{2}}{2}+\int x^{T}a\frac{1}{{\left(2\pi \right)}^{d/2}}\exp\left(-\frac{1}{2}{\left(x-a\right)}^{T}\left(x-a\right)\right)dx\\ \; & =-\frac{{∥a∥}^{2}}{2}+{∥a∥}^{2}=\frac{{∥a∥}^{2}}{2}. \end{array}$$

□

We therefore see that the divergence $D_{2}$ penalises much more strongly the gap between the means of both distributions than the Kullback–Leibler divergence.

The following technical lemma involving the Kullback–Leibler divergence and a change of measure from posterior to prior distribution is pivotal in the PAC-Bayes literature:

**Lemma** **1** ([5,6,7,8,9,10,11,12,13,14,15,16], Corollary 4.15)**.** *Let g be a measurable function g:F↦R such that $\pi \left[e^{g}\right]$ is finite. Let π and ρ be respectively prior and posterior measures as defined in Section 4.1. The following inequality holds:*

$$\rho \left[g\right]\leq \log\pi \left[e^{g}\right]+\mathrm{KL}\left(\rho ,\pi \right).$$

### 4.4. Expensive PAC-Bayesian Bound

The first PAC-Bayesian bound we present is called “expensive PAC-Bayesian bound” in the spirit of Section 2: it is obtained under a sub-Gaussian tails assumption. More precisely, we suppose here that for any f∈F, the distribution of the random variable ℓ(f(X),Y) belongs to $P_{\mathrm{expensive}}^{\sigma }$, which means

$$\log E\left[\exp\left\{\lambda (\ell (f(X),Y)-R(f))\right\}\right]\leq \frac{\lambda^{2}\sigma^{2}}{2},\;\forall \lambda \in R.$$

In this setting, we have the following bound, close to the ones obtained by [10].

**Proposition** **4.** *Assume that for any f∈F, $\ell \left(f\left(X\right),Y\right)\in P_{\mathrm{expensive}}^{\sigma }$. For any prior π, posterior ρ, and any δ∈(0,1), the following inequality holds true with a probability greater than 1−δ:*

$$\rho \left[R\right]\leq \rho \left[R_{N}\right]+\frac{\sigma }{\sqrt{N}}\sqrt{2\left(\log\left(\frac{1}{\delta }\right)+\mathrm{KL}\left(\rho ,\pi \right)\right)}.$$

**Proof.**  The proof is decomposed in two steps. The first leverages Lemma 1. Let λ be a positive number and apply Lemma 1 to the function $\lambda (R-R_{N})$:

$$\rho \left[R\right]\leq \rho \left[R_{N}\right]+\frac{1}{\lambda }\left(\log\pi \left[e^{\lambda (R-R_{N})}\right]+\mathrm{KL}\left(\rho ,\pi \right)\right).$$

The second step is to control the deviations of $\log\pi \left[e^{\lambda (R-R_{N})}\right]$. With a probability 1−δ, we have, by Markov’s inequality

$$\pi \left[e^{\lambda (R-R_{N})}\right]\leq \frac{E\left[\pi \left[e^{\lambda (R-R_{N})}\right]\right]}{\delta }.$$

By Fubini’s theorem, we can exchange the symbols E and π. Using the assumption $P_{\mathrm{expensive}}^{\sigma }$, we obtain with a probability greater than 1−δ

$$\pi \left[e^{\lambda (R-R_{N})}\right]\leq \frac{\exp\left\{\lambda^{2}\sigma^{2}/2N\right\}}{\delta }.$$

Now, putting these results together and setting

$$\lambda =\frac{\sqrt{2N\left(\log\left(\frac{1}{\delta }\right)+\mathrm{KL}\left(\rho ,\pi \right)\right)}}{\sigma }$$

we obtain the desired bound. □

A PAC-Bayesian inequality is a bound which treats the complexity in the following manner:

- At first, a global complexity measure is introduced with the change of measure and is characterised by the divergence term, measuring the price to switch from π (the reference distribution) to ρ (the posterior distribution on which all inference and prediction is based);
- Next, the stochastic assumption on the data-generating distribution is used to control $\pi \left[e^{\lambda (R-R_{N})}\right]$ with high probability.

### 4.5. Cheap PAC-Bayesian Bounds

#### 4.5.1. Using $\chi^{2}$ Divergence

The vast majority of works in the PAC-Bayesian literature focuses on an expensive model. The main reason is that it includes the situation where the loss *ℓ* is bounded, a common (yet debatable) assumption in machine learning. The case where ℓ(f(X,Y) belongs to a cheap model has attracted far less attention; recently, ref. [17] have obtained the following bound.

**Proposition** **5** ([17], Theorem 1)**.** *Assume that for any f∈F, $\ell \left(f\left(X\right),Y\right)\in P_{\mathrm{cheap}}^{\sigma }$. For any prior π, posterior ρ, and any δ∈(0,1), the following inequality holds true with a probability greater than 1−δ*

$$\rho \left[R\right]\leq \rho \left[R_{N}\right]+\frac{\sigma }{\sqrt{N}}\sqrt{\frac{D_{2}\left(\rho ,\pi \right)+1}{\delta }}.$$

The proof (see [17]) uses the same elementary ingredients as in the expensive case, replacing the Kullback–Leibler divergence by $D_{2}$ and the dependence in δ moves from $\sqrt{2\log(1/\delta )}$ to $\frac{1}{\sqrt{\delta }}$. Note the correspondence between these two bounds and the confidence intervals introduced in Section 2.

#### 4.5.2. Using Huber-Type Losses

With a different approach, ref. [18] obtained asymptotic PAC-Bayesian bounds for δ-dependent risk estimators based on the empirical mean of Huber-type influence functions. The author of [18] studied in a slightly more restrictive model than $P_{\mathrm{cheap}}$, assuming in addition that the order 3 moment of ℓ(f(X),Y) is bounded for f∈H. We rephrase here Theorem 9 of [18]: with a probability greater than 1−δ,

$$\rho \left[R\right]\leq \rho \left[{\hat{R}}_{\delta ,N}\right]+\frac{1}{\sqrt{N}}\left(\mathrm{KL}\left(\rho ,\pi \right)+\frac{\log(8\pi \sigma \delta^{-2})}{2}+\sigma +\pi_{N}^{*}\left(F\right)-1\right)+o\left(\frac{1}{N}\right),$$

where $\pi_{N}^{*}\left(F\right)$ is a term depending on the quality of the prior. In Remark 10, the author notes that assuming only finite moments for ℓ(f(X),Y), it is impossible in practice to choose a prior such that $\frac{\pi_{N}^{*}\left(F\right)}{\sqrt{N}}$ decreases at rate $1/\sqrt{N}$ or faster. Then, the dominant term necessarily converges at a slower rate than that of Proposition 4. However, this bounds leads to the definition of a robust PAC-Bayes estimator which proves efficient on simulated data (see Section 5 of [18]).

## 5. A Good Cheap Lunch: Towards a Robust PAC-Bayesian Bound?

If we take a closer look at the aforementioned PAC-Bayesian bounds from a robust statistics perspective, the following question arises: **can we obtain a PAC-Bayesian bound with a $\sqrt{\log(1/\delta )}$ dependence (possibly up to a numerical constant) in the confidence level with the cheap model?** In this section, we shed light on some structural issues. In the following, we assume the existence of σ>0 such that for any f∈F, $\ell \left(f\left(X\right),Y\right)\in P_{\mathrm{cheap}}^{\sigma }$.

### 5.1. A Necessary Condition

Let $\hat{R}$ be an estimator of the risk (not necessarily the classical empirical risk). Here is a prototype of the inequality we are looking for: for any δ∈(0,1), with probability 1−δ

$$\rho \left[R\right]\leq \rho \left[\hat{R}\right]+\frac{\sigma }{\sqrt{N}}A\left(\rho ,\pi ,\delta \right),$$

where

$$A\left(\rho ,\pi ,\delta \right)\underset{\delta \to 0}{=}O\left(\sqrt{\log(1/\delta )}\right).$$

If we choose $\rho =\pi =\delta_{\left\{f\right\}}$ (Dirac mass in the single hypothesis *f*), the existence of such a PAC-Bayesian bound valid for all δ implies that

$$\left[\hat{R}\left(f\right)\pm \frac{\sigma }{\sqrt{N}}\times c\sqrt{\log(1/\delta )}\right]$$

is a confidence interval for the risk R(f) for any level 1−δ, where *c* is a constant.

Thus, a necessary condition for a PAC-Bayesian bound to be valid for all of the risk level δ is to have tight confidence intervals for any f∈F.

However, as covered in Section 3, such estimators do not exist over the class $P_{\mathrm{cheap}}^{\sigma }$, and the possibility to derive a tight confidence interval is limited by the fact that the level δ must be greater that a positive constant of the form $e^{-O(N)}$.

### 5.2. A δ-Dependent PAC-Bayesian Bound?

As a consequence, there is simply no hope for a robust PAC-Bayesian bound valid for any error threshold δ, for essentially the same reason which prevents it in the mean estimation case. The question we address now is the possibility of obtaining a robust PAC-Bayesian bound, with a dependence of magnitude $\sqrt{2\log(1/\delta )}$ (possibly up to a constant), with a possible limitation on the error threshold δ. In the following, we assume to have an estimator of the risk $\hat{R}$ and an error threshold δ>0 such that there exists a constant C>0 such that for any f∈F,

$$\left[\hat{R}\left(f\right)\pm \frac{\sigma }{\sqrt{N}}\times C\;\sqrt{\log(1/\delta )}\right]$$

is a confidence interval for R(f) at level 1−δ. MoM is an example of such estimator. Let us stress that δ is fixed and cannot be used as a free parameter.

As seen above, a PAC-Bayesian bound proof proceeds in two steps:

- First, we use a convexity argument to control the target quantity $\rho [R-\hat{R}]$ by an upper-bound involving a divergence term and a term of the form $g^{-1}\left(\pi \left[g(R-\hat{R})\right]\right)$ where *g* is a non-negative, increasing, and convex function;
- Second, we control the term $\pi \left[g(R-\hat{R})\right]$ in high probability, using Markov’s inequality.

The first step does not require any use of a stochastic model on the data, and is always valid, regardless of whether we have a cheap or an expensive model. The second step uses the model and introduce the dependence in the error rate δ on the right-term of the bound: $g^{-1}\left(1/\delta \right)$. In the case of the “expensive bound”, we had g=exp, and the dependence was log(1/δ), the final rate $\sqrt{\log(1/\delta )}$ was obtained by choosing a relevant value for λ.

Let us follow this scheme to obtain a robust PAC-Bayesian bound. The first step gives

$$\rho \left[R\right]\leq \rho \left[\hat{R}\right]+\frac{1}{\lambda }\left(\log\pi \left[e^{\lambda (R-\hat{R})}\right]+\mathrm{KL}\left(\rho ,\pi \right)\right).$$

Our goal is now to control $\pi \left[e^{\lambda (R-\hat{R})}\right]$ in high probability.

#### 5.2.1. The Case $\pi =\delta_{\left\{f\right\}}$

Let us start with a very special case, where the prior is a Dirac mass on some hypothesis f∈F. Then

$$\frac{1}{\lambda }\log\pi \left[e^{\lambda (R-\hat{R})}\right]=R\left(f\right)-\hat{R}\left(f\right).$$

Using how $\hat{R}$ is defined, we can bound this quantity in the following way: with probability 1−δ,

$$R\left(f\right)-\hat{R}\left(f\right)\leq \frac{\sigma }{\sqrt{N}}\times C\sqrt{\log(1/\delta )}.$$

Another way to formulate this result is to say that there exists an event $A_{f}$ with a probability greater than 1−δ such that for all $\omega \in A_{f}$, the following holds true:

$$\left(R\left(f\right)-\hat{R}\left(f,\omega \right)\right)\leq \frac{\sigma }{\sqrt{N}}\times C\sqrt{2\log(1/\delta )}.$$

In this example, we can control $\log\pi \left[e^{\lambda (R-\hat{R})}\right]$ at the price of a maximal constraint on the choice of the posterior. Indeed, the only possible choice for ρ for the Kullback–Leibler KL(ρ,π) to make sense is $\rho =\pi =\delta_{\left\{f\right\}}$.

#### 5.2.2. The Case $\pi =\alpha \delta_{\left\{f_{1}\right\}}+\left(1-\alpha \right)\delta_{\left\{f_{2}\right\}}$

Consider now a somewhat more sophisticated choice of prior which is a mixture of two Dirac masses in two distinct hypotheses. We do not fix the mixing proportion α and allow it to move freely between 0 and 1. The goal is to control the quantity

$$\pi \left[e^{\lambda (R-\hat{R})}\right]=\alpha e^{\lambda (R\left(f_{1}\right)-\hat{R}\left(f_{1}\right))}+\left(1-\alpha \right)e^{\lambda (R\left(f_{2}\right)-\hat{R}\left(f_{2}\right))}.$$

More precisely, for all α∈(0,1), we want to find an event $A_{\alpha }$ on which this quantity is under control. In view of the prior’s structure, the only way to ensure such a control is to have $A_{\alpha }\subset A_{f_{2}}\cap A_{f_{2}}$, where $A_{f_{1}}$ (resp. $A_{f_{2}}$) is the favourable event for the concentration of $\hat{f_{1}}$ (resp. $\hat{f_{2}}$) around its mean.

By the union bound, we have that with a probability greater than 1−2δ

$$\frac{1}{\lambda }\log\pi \left[e^{\lambda (R-\hat{R})}\right]\leq \frac{\sigma }{\sqrt{N}}\times C\sqrt{\log(1/\delta )}.$$

We face a double problem here. As above, if we want the final bound to be non-vacuous, we have to ensure that KL(ρ,π) is finite, which restricts the support for the posterior to be included in the set $\left\{f_{1},f_{2}\right\}$. In addition, the PAC-Bayesian bound holds with a probability greater than 1−2δ…

#### 5.2.3. Limitation

… which hints at the fact that this will become 1−Kδ if the support for the prior contains *K* distinct hypotheses. If K≥1/δ, the bound becomes vacuous. In particular, we cannot obtain a relevant bound using this approach in the situation where the cardinal of F is infinite (which is commonly the case in most PAC-Bayes works).

This limiting fact highlights that to derive PAC-Bayesian bounds, we cannot rely on the construction of confidence interval for all R(f) for a fixed error threshold δ. The issue is that when we want to transfer this local property into a global one (valid for any mixture of hypotheses by the prior π), we cannot avoid a worst-case reasoning by the use of the union bound.

The established bounds in the PAC-Bayesian literature, both in cheap and expensive models, repeatedly use the fact that when we assume that for any f∈F,

$$\log E\left[e^{\lambda (R(f)-\ell (f(X),Y))}\right]\leq \frac{\lambda^{2}\sigma^{2}}{2},\;\forall \lambda \in R$$

or

$$\mathrm{var}\left(\ell (f(X),Y)\right)\leq \sigma^{2},$$

we make an implicit assumption on the integrability of the tail of the distribution of ℓ(f(X),Y). This argument is crucial for the second step of the PAC-Bayesian proof because, by Fubini’s theorem, it allows us to convert a local property (the tail distribution of each ℓ(f(X),Y)) into a global one (the control of $\pi \left[e^{\lambda (R-R_{N})}\right]$ or $\pi \left[\left(R-R_{N}\right){)}^{2}\right]$ in high probability).

### 5.3. Is That the End of the Story?

We have identified a structural limitation to derive a tight PAC-Bayesian bound in a cheap model. We make the case that we cannot replicate the PAC-Bayesian proof presented in Section 4. To conclude this section, we want to highlight the fact that, up to our knowledge, no proof of PAC-Bayesian bounds avoids these two steps (see, for example, the general presentation in [19]).

What if we try to avoid the change of the measure step and try to control directly $\rho \left[R\right]-\rho \left[\hat{R}\right]$ in high probability? We remark that ρ can only be chosen with the information given by the observation of $\hat{R}\left(f\right)$, where f∈F. In particular, we cannot obtain any information of the concentration of each $\hat{R}\left(f\right)$ around R(f) as such knowledge requires to know the true risk. So, it seems that a direct control cannot avoid starting as a “worst-case” bound:

$$\rho \left[R\right]-\rho \left[\hat{R}\right]\leq \underset{f\in F}{\sup}\left\{R\left(f\right)-\hat{R}\left(f\right)\right\}.$$

Then, we have to control $\sup_{f\in F}\left\{R\left(f\right)-\hat{R}\left(f\right)\right\}$ in high probability (see [20] for a general presentation on such controls, and [7] for the recent results in the special case where $\hat{R}$ is a MoM estimator). However, the obtained bound will take the following prototypic form:

$$\rho \left[R\right]\leq \rho \left[\hat{R}\right]+\mathrm{complexity}\;\mathrm{term},$$

where the complexity term does not depend on the distribution ρ. Thus, the optimisation of the right term leads to choosing ρ as the Dirac mass in $\underset{f\in F}{\arg\min}\;\hat{R}\left(f\right)$.

So, the overall procedure amounts to a slightly modified empirical risk minimisation (where the empirical mean is replaced with any estimator of the risk), and will not fall into the category of generalised Bayesian approaches which take into account the uncertainty on hypotheses. Pretty much all the strengths of PAC-Bayes would then be lost.

## 6. Conclusions

The present paper contributes a better understanding of the profound structural reasons why good cheap lunches (tight bounds under minimal assumptions) are not possible with PAC-Bayes by walking gently through elementary examples.

From a theoretical perspective, PAC-Bayesian bounds requires too strong assumptions to adapt robust statistics results (where almost good lunches can be obtained for cheap models—with the limitation that the confidence level is constrained). The second step of the proof we have shown requires us to transform a local hypothesis, a control of some moments of ℓ(f(X),Y), into a global one, valid for all mixture of hypotheses by the prior π. As covered above, this transformation seems impossible.

To close on a more positive note after this negative result, let us stress that even if the conciliation of PAC-Bayes and robust statistics appears challenging, we believe that the recent ideas from robust statistics could be used in practical algorithms inspired by PAC-Bayes. In particular, we leave as an avenue for future work the empirical study of PAC-Bayesian posteriors (such as the Gibbs measure defined as $\rho \propto \exp(-\gamma \hat{R})\pi$ for any inverse temperature γ>0) where the risk estimator is not the empirical mean (as in most PAC-Bayes works) but rather a robust estimator, such as MoM.

## Data Availability

Not applicable.
